# A Novel Loss-of-Function Variant in the Chloride Ion Channel Gene *Clcn2* Associates with Atrial Fibrillation

**DOI:** 10.1038/s41598-020-58475-9

**Published:** 2020-01-29

**Authors:** Thea Hyttel Hansen, Yannan Yan, Gustav Ahlberg, Oliver Bundgaard Vad, Lena Refsgaard, Joana Larupa dos Santos, Nancy Mutsaers, Jesper Hastrup Svendsen, Morten Salling Olesen, Bo Hjorth Bentzen, Nicole Schmitt

**Affiliations:** 1 Department of Biomedical Sciences, Faculty of Health and Medical Sciences, University of Copenhagen, Copenhagen, Denmark; 2 Laboratory for Molecular Cardiology, Department of Cardiology, The Heart Centre, Righospitalet, Copenhagen University Hospital, Copenhagen, Denmark; 3 Department of Clinical Medicine, Faculty of Health and Medical Sciences, University of Copenhagen, Copenhagen, Denmark; 4 Present Address: ALK-Abelló A/S, 2970 Hørsholm, Denmark

**Keywords:** Atrial fibrillation, Cardiovascular genetics

## Abstract

Atrial Fibrillation (AF) is the most common cardiac arrhythmia. Its pathogenesis is complex and poorly understood. Whole exome sequencing of Danish families with AF revealed a novel four nucleotide deletion c.1041_1044del in *CLCN2* shared by affected individuals. We aimed to investigate the role of genetic variation of *CLCN2* encoding the inwardly rectifying chloride channel ClC-2 as a risk factor for the development of familiar AF. The effect of the *CLCN2* variant was evaluated by electrophysiological recordings on transiently transfected cells. We used quantitative PCR to assess *CLCN2* mRNA expression levels in human atrial and ventricular tissue samples. The nucleotide deletion *CLCN2* c.1041_1044del results in a frame-shift and premature stop codon. The truncated ClC-2 p.V347fs channel does not conduct current. Co-expression with wild-type ClC-2, imitating the heterozygote state of the patients, resulted in a 50% reduction in macroscopic current, suggesting an inability of truncated ClC-2 protein to form channel complexes with wild type channel subunits. Quantitative PCR experiments using human heart tissue from healthy donors demonstrated that *CLCN2* is expressed across all four heart chambers. Our genetic and functional data points to a possible link between loss of ClC-2 function and an increased risk of developing AF.

## Introduction

Atrial fibrillation (AF) is the most common form of cardiac arrhythmia. In the general population, it is estimated that 2–3% are affected^1–3^. Prevalence increases significantly with age and more than 10% of the population aged 80 years or older are estimated to have AF^2,3^. Early-onset of the disease is commonly defined as onset before the age of 65^4–6^. The disease is characterized by rapid and uncoordinated electrical activation of the atria that prevents a concerted contraction of the atrial myocardium^1^. This can cause reduced ventricular filling and blood stasis in the atria, which is believed to predispose for heart failure and thromboembolic stroke. In epidemiological studies, AF has also been associated with increased morbidity and mortality^1^. A large fraction of AF patients have comorbidities or carry risk factors for thromboembolic complications such as hypertension, diabetes, valvular heart disease, and heart failure^7^. AF patients devoid of comorbidities, i.e. evidence of other cardiovascular or pulmonary diseases, are referred to as ‘lone AF’ patients. This subgroup tends to be younger at onset of AF. In addition, a family history of AF is also more commonly seen among these patients indicating that ‘lone’ AF patients carry a greater genetic predisposition for the disease than the general AF patient^8^. Studying the genetic background of these patients therefore provides an opportunity for gaining additional insight into the complex pathophysiology of AF.

Here we describe a Danish family with early onset AF. Using whole exome sequencing (WES), we identified a novel loss-of-function variant in the gene *CLCN2* that co-segregated with affected family members. *CLCN2* encodes the inwardly rectifying chloride (Cl^−^) channel ClC-2 that is activated upon membrane hyperpolarization, cell swelling and acidosis^9^. *CLCN2* loss-of-function mutations have so far been linked to leukoencephalopathies^10^ and azoospermia^11^, while gain-of-function mutations are found in families with hyperaldosteronism type 2^12,13^. Although a Cl^−^ channel with properties resembling ClC-2 has been recorded in atrial and ventricular cardiomyocytes from different mammals, and ClC-2 has been found to be important for sinus nodal pacemaker activity^14–17^, the precise role of ClC-2 in the heart is not known.

## Results

### Clinical characteristics

**Figure 1:**
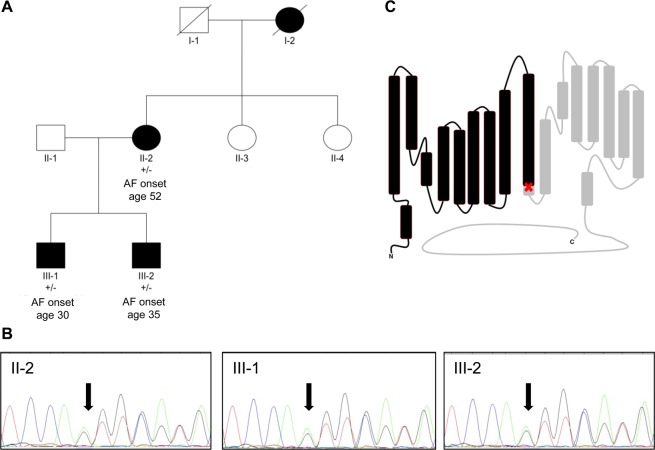
Genetic information. (**A**) Pedigree of the family with *CLCN2-* c.1041_1044del variant. Square: Male. Circle: Female. Black filled for AF affected individual. White filled for unaffected individual. Diagonal line for diseased individual. The presence of the variant is indicated with “+” for presence and “−” for absence. (**B**) Sanger chromatograms of the affected patients. (**C**) Schematic presentation of ClC-2 protein topology indicating position of frame shift mutation (red cross) and effect of mutation (truncated part in grey).

We identified a family with three living family members affected by AF, and one deceased family member with a history of AF (Fig. 1A). Clinical features of the affected family members are provided as Supplementary Table S1. All affected family members were included, and all were found to carry the c.1041_1044delGGTG variant in *CLCN2* (Fig. 1B). Material from non-affected family members was not available.

The proband (III-1) had onset of paroxysmal AF at age 30, and pharmacological treatment was initiated with beta-blockers. He had normal blood pressure and no other comorbidities. Due to increasing frequency and lengths of AF episodes, he underwent two radio frequency ablations which reduced the frequency of episodes to three to four times per year. His echocardiogram showed a small central mitral regurgitation, with a normal left ventricular function and no signs of structural heart disease, and a heart CT-scan found normal coronary arteries.

The probands brother (III-2) had onset of symptoms at age 32 and was diagnosed with AF at age 35. He was treated with beta-blockers and electrical cardioversion was performed four times due to persistent AF. He was overweight at the time of disease onset, and was diagnosed with, and treated for thyrotoxicosis two years after his onset of AF. His blood pressure was normal, and echocardiogram showed a normal function of all chambers, with no signs of structural heart disease.

Their mother (II-2) had onset of symptoms at age 30 and was diagnosed with AF at age 52. Antiarrhythmic treatment was attempted with the Na^+^-channel blocker Flecainide and Beta-blockers, however over the following 14 years the AF became permanent. No comorbidities were present at diagnosis of AF; however the subject later suffered from ischemic stroke, hypertension and chronic obstructive lung disease. Echocardiographic examination was performed and found enlarged left atria, and otherwise normal cardiac structure and function.

### Genetic variation

WES was performed on three affected family members in parallel (II-2, III-1, III-2; Fig. 1). Sequencing generated a mean coverage of 95 reads (Supplementary Tables S2). More than 97.8% of targeted bases were covered with >10 reads and more than 93.9% of target bases >20 reads. The genetic analysis was aimed at identifying rare or novel protein altering variants shared by affected family members. We identified 18 rare variants that meet the criteria (Supplementary Tables S3–S4). Of these variants, a frame shift variant in *CLCN2* had the most protein damaging impact, with a CADD PHRED score of 34. We performed pathway analysis of those variants with similar minor allele frequency in the Danish population (Supplementary Figs. S1–S5). Pathway analysis indicated that the protein product of *CLCN2* might interact with several other proteins that could be involved in AF^18^.

*CLCN2* encodes the chloride channel called ClC-2. While the full-length protein consists of 899 amino acids, the four nucleotide deletion c.1041_1044delGGTG and resulting frame shift leads to a truncated channel protein with the last common amino acid glutamine 347 followed by eleven amino acids before a premature stop (p.Val347fs, ENST00000265593), thereby disrupting a highly conserved area of the protein (Fig. 1C and Supplementary Fig. S6).

### *CLCN2* mRNA is expressed in human atria

**Figure 2:**
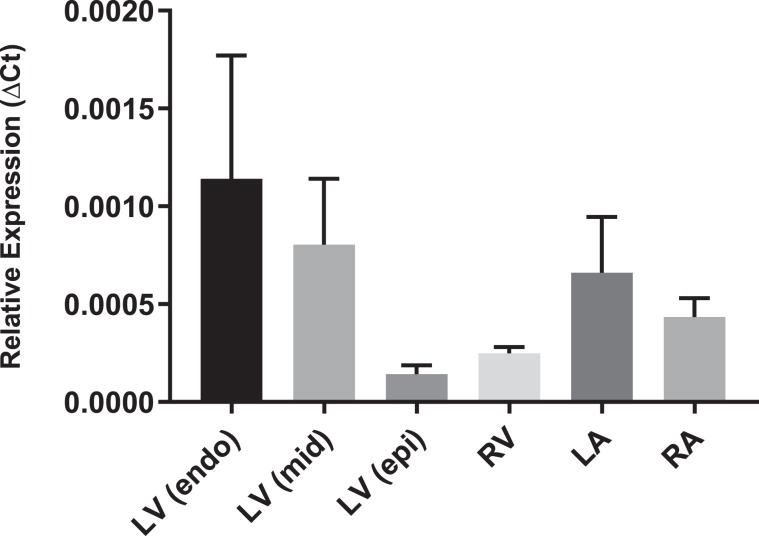
Relative expression of *CLCN2* in tissue from the four heart chambers of healthy individuals (n = 7) analyzed by qPCR. *YWHAZ* and *RPL13A* were used as reference genes. LV – left ventricle; Endo - endocardium; myo - myocardium; epi – epicardium; RV – right ventricle; LA – left atrium; RA – right atrium.

We assessed expression levels of *CLCN2* in human heart by quantifying mRNA levels using qPCR (replicates n = 7; right atrium, left atrium, right ventricle and left ventricle, endocardium, myocardium and epicardium). *CLCN2* transcripts were detected in all chambers and across the left ventricular wall, with lower expression in the left epicardium and right ventricle (Fig. 2). There was no significant difference in expression levels between the heart chambers.

Furthermore, we tested whether induced pluripotent stem cell derived cardiomyocytes (iPSC-CM) would be a suitable tool to study the role of the ClC-2 variant. To this end, we assessed ClC-2 expression in 32 and 203 day old control iPSC-CM by reverse transcription PCR. However, we did not observe ClC-2 expression in these samples (Supplementary Fig. S7).

### The *CLCN2* truncation mutant exhibits a loss-of-function phenotype

**Figure 3:**
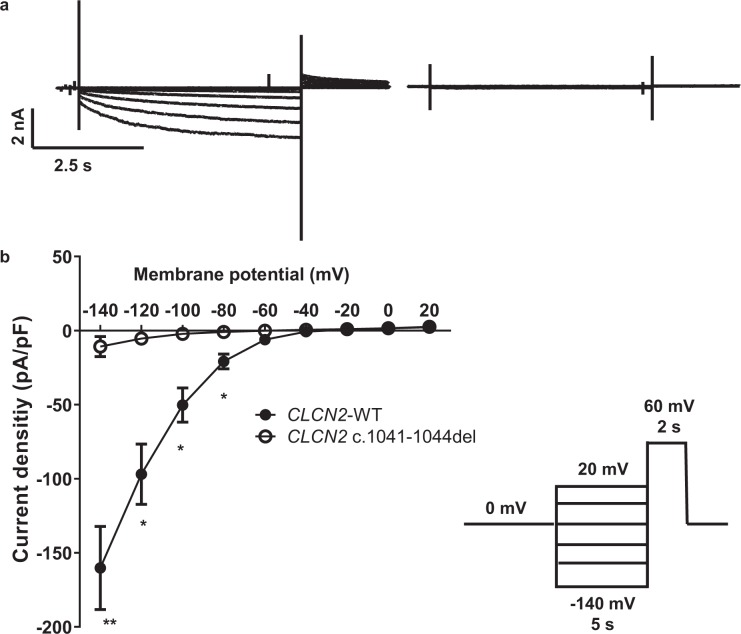
Effect of ClC-2 variant on whole cell current. (**a**) Representative current traces of whole-cell voltage-clamp recordings from HEK293 cells transiently transfected with ClC-2 WT (left) or mutated ClC-2 (*CLCN2*- c.1041_1044del) (right). Currents were elicited by the voltage protocol shown. (**b**) Current voltage relationship of wild type (closed circles) and mutated ClC-2 (open circles), n = 9 and 10.

To address the functional effect of the ClC-2 truncation mutant, we expressed wild-type (WT) and mutant ClC-2 channels in Human Embryonic Kidney (HEK)293 cells and assessed channel function by patch-clamp electrophysiology. In line with literature^19–21^, ClC-2 WT showed slow activation at hyperpolarized potentials, no time-dependent inactivation and an inwardly rectifying current-voltage relationship (Fig. 3). In contrast, no current could be elicited in HEK293 cells expressing ClC-2 p.V347fs, suggesting that the frame shift mutation causes a complete loss-of-function of the ClC-2 channel.

**Figure 4:**
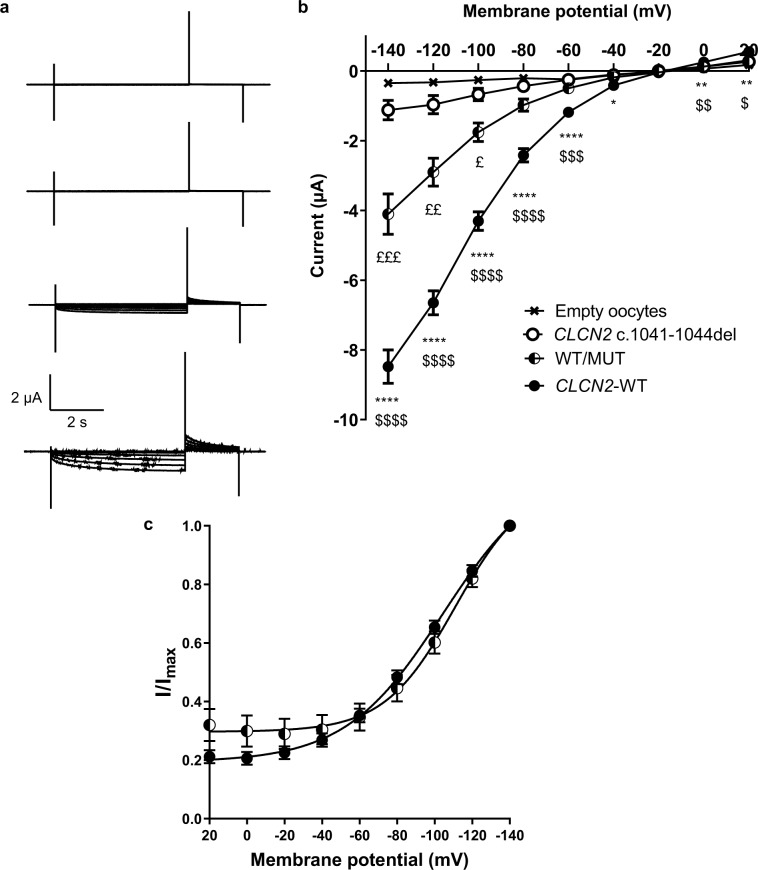
Effect of heterozygous expression of ClC-2 variant. (**a**) Representative current traces from empty *Xenopus laevis* oocytes and oocytes expressing ClC-2 WT, ClC-2 MUT or ClC-2-WT and ClC-2-MUT co-expressed (50% + 50%). Currents were elicited by the voltage protocol shown in the inset. (**b**) Current voltage relationship of empty oocytes (crosses), wild type (closed circles), mutated ClC-2 (open circles), and heterozygote expression (half-filled) n = 18, 40, 30, and 19, respectively). (**c**) Voltage-dependence of channel activation relationships from oocytes expressing wild type or co-expressing wild type and mutated channels. ^*^WT vs. MUT; ^$^WT vs. WT/MUT; ^£^WT/MUT vs. MUT.

To mimic the heterozygous state of the patients and thereby address whether the variant may exert a dominant negative effect or show a haploinsufficient trait, we took advantage of the *Xenopus laevis* oocyte expression system that allows for controlled co-expression of ion channel subunits. The different cRNAs are directly injected into the oocytes, thereby bypassing the inherent problems of performing co-expression studies using lipid-based transfection of mammalian cells. We verified that the effect of the ClC-2 variant was consistent between the oocyte and the HEK293 models (Fig. 3). Recordings from oocytes expressing ClC-2 WT gave rise to hyperpolarization activated inwardly rectifying currents, whereas oocytes expressing the mutated channel had current amplitudes that were not significantly different from empty oocytes (Fig. 4a,b). Co-expression of mutant and WT ClC-2 imitating the heterozygous state of the patient (50% WT and 50% mutant) resulted in significant current reduction as compared to CLC-2 WT, and current amplitudes of approximately 50% of the WT current. Moreover, the voltage-dependence of activation was not changed between oocytes expressing wild type channels or co-expressing both WT and mutant channels (Fig. 4c).

### Protein expression of the ClC-2 mutant is decreased

**Figure 5:**
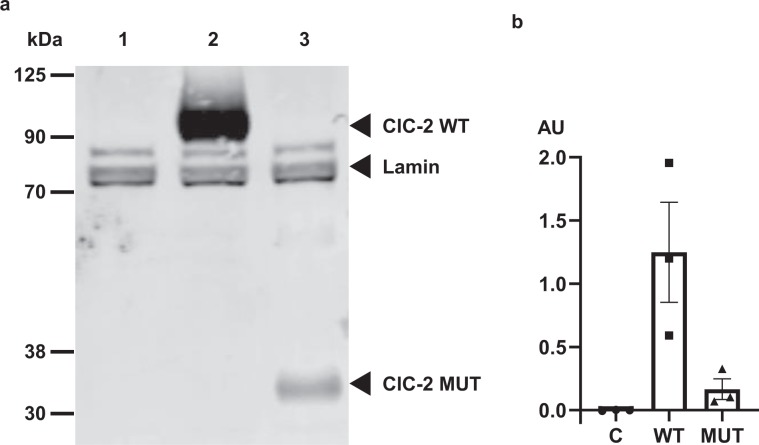
Western blot of HEK293 cells expressing WT or mutant channels shows lowered protein levels. (**a**) Representative western blot of protein extracts from HEK293 cells transfected with empty plasmid (lane 1), ClC-2 WT (lane 2) or ClC-2 MUT (lane 3). Lamin was used as a loading control. Bands representing ClC-2 or Lamin proteins are indicated by arrows. (**b**) Quantification of protein expression from three independent experiments.

The nucleotide deletion c.1041_1044delGGTG introduces a premature stop codon that results in a truncation of the protein after amino acid 347. We investigated the effect of the mutation on channel protein expression (Fig. 5). We employed N-terminally HA-tagged constructs to enable visualization of both WT and truncated ClC-2. Western blotting analysis of ClC-2 WT showed a band at the expected apparent molecular weight of approximately 90 kDa. The mutant ClC-2 p.V347fs appeared as a protein band with an estimated molecular weight of approximately 35 kDa (Fig. 5a, Supplementary Fig. S8). Protein expression of ClC-2-V347fs was significantly decreased compared to ClC-2 WT (Fig. 5b).

## Discussion

In a Danish family spanning three generations with early onset AF, employing whole exome sequencing we identified a novel frame shift variant in *CLCN2* that co-segregated with the disease. We established that *CLCN2* mRNA was present in all four chambers of the human heart.

*CLCN2* encodes the chloride ion channel ClC-2 originally cloned from rat heart and brain^19^. ClC-2 subunits comprise 16 transmembrane segments B-Q with intracellular N- and C-termini. Two subunits form a homodimer, with a protopore in each subunit^22–24^. The frame-shift mutation leads to a premature stop that truncates the ClC-2 protein between the predicted transmembrane helices J and K^24^. Western blot analysis revealed that mammalian cells can produce the truncated protein ClC-2-V347fs, albeit at a markedly decreased abundance.

Patch-clamp experiments revealed that the truncated ClC-2 channel does not conduct any current. When co-expressed with ClC-2 WT, imitating the heterozygous state of the patients, a 50% reduction in macroscopic current was recorded. Hence, the mutant channel subunits did neither exert a dominant effect on the WT channel nor did WT channel subunits rescue the mutant phenotype, indicative of haploinsufficiency in the patients. This suggests that the truncated ClC-2 protein is not able to form channel dimers with wild-type channel subunits.

To our knowledge, this is the first time that a variant in *CLCN2* has been associated with cardiac disease. So far genetic loss-of-function mutations have been found in patients with leukoencephalopathies with symptoms including ataxia, visual field defects, and learning disabilities^10^ and azoospermia^11^, which resembles the phenotype of in *Clcn2*-deficient mice^25,26^. The available patient records did not disclose leukoencephalopathies or azoospermia in the male patients. Whereas we cannot rule out that the heterozygous patients are undiagnosed or will develop other phenotypes at later age, heterozygous *Clcn2* knock-out mice (ClC-2^+^/_−_) did not exhibit morphological or functional CNS phenotypes^26^.

ClC-2 is ubiquitously expressed in both epithelial and non-epithelial tissue and *CLCN2* gain-of-function mutations have been recently identified in several families with familial hyperaldosteronism type 2^12,13^. ClC-2 currents have been recorded in different glandular and ductal cells, testicular cells, erythrocytes, rat retinal bipolar cells, and other CNS cell types, including sympathetic neurons, and carotid body chemoreceptor cells^27,28^. Less is known about ClC-2’s role in the heart. However, replacing chloride in the extracellular solution with impermeable anions increases ventricular action potential duration and maximal diastolic potentials^29^. The concentration of intracellular Cl^−^ in ventricular cardiomyocytes is about 10–20 mM due to the activity of different electroneutral co-transporters. This results in an equilibrium potential of Cl^−^ in cardiomyocytes of around −65 to −45 mV (for review see^30^). Using electrophysiological and molecular biological techniques Duan *et al*. identified an inward rectifying Cl^−^ current in atrial and ventricular cardiomyocytes from mouse and guinea pig that was activated by membrane hyperpolarization (−40 to −140 mV) and hypotonic cell swelling. These properties are similar to the currents generated by expression of ClC-2 in heterologous expression systems. Together with data confirming expression at the mRNA level, the authors suggested that the inwardly rectifying Cl^−^ current recorded in cardiomyocytes is encoded by *CLCN2*^14^. These results have been corroborated in several other studies using different species or physiological triggers^15,16,31,32^. However, the functional effect of ClC-2 on action potential generation and conduction in cardiomyocytes has not been investigated, likely because of the lack of ClC-2 specific pharmacological modulators. Leiuropeptide II (also called GaTx2) has been proposed as selective ClC-2 blocker. The toxin slowed ClC-2 activation and inhibited slow-gating, but did not inhibit open ClC-2 channels^33^. However, it has been pointed out that the GaTx2 has not been independently verified in other studies^9^. In our hands, GaTx2 did not inhibit heterologously expressed ClC-2 channels (data not shown). A role of ClC-2 in cardiac pacemaker activity has also been explored. In guinea pig sinus nodal cells an inward rectifying Cl current with properties matching the ClC-2 channel has been recorded^17^. Hypotonic stress depolarized the maximum diastolic potential, increased the slope of the diastolic membrane potential and shortened the action potential duration. These effects were somehow reversed by intracellular dialysis with anti-ClC-2 antibodies^17^. Moreover, telemetry ECG recordings on conscious ClC-2 knockout mice showed that the chronotropic response to exercise was reduced in the KO mice as compared to littermate control mice. Together with the lack of effect on intrinsic heart rate the authors concluded that ClC-2 may play an important role for pacemaker activity especially under sympathetic stimulation^17^.

Overall, the reduction in CLC-2-V347fs protein production and the lack of a dominant negative effect indicates that the truncated protein is not contributing to dimer formation, and hence likely results in haploinsuffiency. Taken together with previous findings of ClC-2 in mammalian pacemaker activity, cardiac action potential generation, and the expression of *CLCN2* in the human atria, this suggests that variation in *CLCN2* could predispose for AF.

## Limitations

Our findings are based on cellular assays. As we did not detect ClC-2 expression in 32 or 203 days iPSC-derived cardiomyocytes, we were unable to investigate the possible effects in this model system. Due to poor pharmacology of CLC-2^9^, we were unable to test arrhythmogenicity in *ex vivo* or *in vivo* settings in animals. We refrained from studying available transgenic mouse models as mice are considered less suitable in arrhythmia research^34^.

## Conclusion

Using WES, we identified a novel frame-shift variant in *CLCN2* that co-segregated with early onset AF and that displays a loss-of-function phenotype upon electrophysiological analysis. We show that *CLCN2* is expressed in both human atria. Previous reports on ClC-2’s role in cardiac electrophysiology and our findings indicate that genetic variation in the voltage-gated chloride channels ClC-2 might predispose to AF.

## Methods

All experiments were performed in accordance with relevant guidelines and regulations.

### Research subjects

All study participants provided written informed consent. The study was approved by the scientific ethics committee for the Capital Region of Denmark (protocol number H-1-2011-044) and performed in accordance with national guidelines. Blood samples was drawn from participating family members and used for whole-exome sequencing. Clinical information was obtained through a questionnaire and review of patient health records.

AF was defined as a discharge diagnosis in the presence of the International Classification of Diseases (ICD)-8 code 427.93, 427.94, and ICD-10 code I48. Details on inclusion of family members have been described previously^35^.

### Genetic analysis

Exome sequencing and bioinformatics have been described previously^35^. In brief, samples were prepared from peripheral blood using the Maxwell 16 LEV Blood DNA Kit. Exome DNA was sequenced on an Illumina HiSeq. 2500 machine using Broad Institute (BI) bait capture kit (38 Mb target region). After trimming of adapter sequences and filtering reads, alignment was made to the human reference genome (NCBI Build 37) and followed by post-processing according to the Genome Analysis Toolkit version 3.4 (GATK) guidelines^36^. Variants were called with Haplotypecaller/GATK v3.4. Genotypes were quality controlled and filtered according to GATK guidelines. Family relatedness was inferred with the King robust algorithm with an LD threshold of 0.5, using R package SnpRelate^37^.

The genetic analysis aimed at identifying rare (ExAC minor allele frequency [MAF] <0.01% and Allele count <=1 in 2000 Danish exomes) or novel protein altering variant, which cosegregated with the disease in the family. Rare variants were defined from MAF in ExAC, dbSNP b.142 and an in-house Danish population of 1,972 exomes^38–40^. Identified variants were prioritized by predicted severity of impact on protein function, using CADD v1.4 scores^41^. Potentially pathogenic variants are normally considered more likely to be found in variants with a PHRED score over 20. The CADD PHRED-scale variants represent rank rather than magnitude, e.g. PHRED score 10–20, top 1% and PHRED score 20–30, top 0.1% and so on.

### Cloning

The mammalian expression construct pcDNA3.1V5His carrying human *CLCN2* cDNA (GenBank Accession Number NM_004366) was a kind gift from Dr. Christel Depienne (Pierre-and-Marie-Curie University, Paris, France) and has been described previously^42^. For expression in *Xenopus laevis* oocytes, we amplified the *CLCN2* cDNA from the pcDNA3.1V5His construct using primers with engineered restrictions sites and cloned the amplicon into the vector pGemHE using BamHI and XbaI sites. The mutation c.1041_1044delGGTG (p.V347fsTer11) was introduced by mutated oligonucleotide extension (PfuTurbo Polymerase, Agilent Technologies, Santa Clara, California) with primers 5′-GAACCGGAAGATTGTCCAATGCGGAAGCAGAAAAC-3′ (forward) and 5′-GTTTTCTGCTTCCGCATTGGACAATCTTCCGGTTC-3′ (reverse). Available ClC-2 antibodies recognize the C-terminal region of the channel and are hence not suitable for analysis of C-terminally truncated channels. Instead, we engineered an N-terminal HA-tag to *CLCN2* wild-type or mutant cDNA using standard PCR techniques. All plasmids were verified by complete sequencing of the cDNA insert (Macrogen Inc., Amsterdam, The Netherlands).

### Quantitative PCR expression analysis

Cardiac biopsies were from healthy donor hearts deemed unsuitable for transplantation and obtained with appropriate consent. The biopsies were snap-frozen in liquid nitrogen immediately after dissection and stored at −80 °C until further use. The anonymized samples had been provided by Dr. András Varró (University of Szegeb, Hungary) under ethical approval number 4991-0/2010-1018EKU (339/PI/010). We used biopsies from seven individuals. Five were females between 17–54 years old and two males that were 21 and 44 years old. Left ventricle biopsies were separated into endo-, myo- and epicardium. The preparation of these samples has been described in detail previously^43^.

For each tissue, 40 mg of biopsy was homogenized in QIAzol reagent (QIAGEN, Maryland, USA) with a Precellys 24 homogenizer (Bertin Technologies, Montigny-le-Bretonneux, France). Total RNA was purified following manufacturer’s instructions (miRNeasy Mini kit, QIAGEN, Hilden, Germany). Genomic DNA contamination was removed with DNAse and RNA concentration and purity was assessed by spectrophotometry (NanoDrop2000, ThermoScientific, Wilmington, USA). Polyadenylated RNAs were reverse-transcribed to cDNA (Nanoscript2 Reverse Transcription kit, Primerdesign, United Kingdom) according to manufacturer’s instructions. The expression of *CLCN2* was measured with quantitative polymerase chain reactions (qPCR) in triplicates, using Taqman double dye probes and PrecisionPLUS MasterMix with ROX (Primerdesign, United Kingdom) in a CFX Connect Real-Time System (BIO-RAD, Hertfordshire, UK). Primer sequences were 5′-CCGCTGCCACAAGTTCCTA-3′ (forward) and 5′-GCTGACCAATGCCATGAGAAG-3′ (reverse). The following qPCR steps were used: 95** °**C for 2 min followed by 50 cycles of 95 °C for 15 s and 60 °C for 1 min. As a reference to normalize the data, we used *YWHAZ* (Ref. NM_003406, PrimerDesign, UK) and *RPL13A* (Ref. NM_012423, PrimerDesign, UK). Threshold cycle (C*t*) values were transferred to a spreadsheet for calculation of ΔC*t*s and the relative expression was calculated using the 2^−ΔCt^ method.

### Cell culture and transfection

HEK293 cells (Sigma-Aldrich, Brøndby, Denmark) were cultured in Dulbecco’s Modified Eagle’s Medium (DMEM, in-house, University of Copenhagen, Denmark) supplemented with 10% fetal bovine serum and 1% penicillin/streptomycin (Thermo Fisher Scientific, Waltham Massachusetts, USA) and maintained in a humidified atmosphere with 5% CO_2_ at 37 °C. HEK293 cells were transiently transfected with 0.3 μg plasmid carrying WT or mutant ClC-2 and 0.1 µg plasmid carrying enhanced green fluorescent protein (eGFP) when 70–90% confluent in T25 culture flasks using SilentFect (Bio-Rad, Copenhagen, Denmark).

Two days after transfection, cells were trypsinized and seeded on coverslips (Menzel Gläser, Ø 3.5 mm, Thermo Scientific) for electrophysiological experiments. For Western blots experiments, HEK293 cells were transfected with 1 μg plasmid carrying HA-tagged WT or mutant ClC-2 and harvested 24 h after transfection.

### Patch clamp experiments

Whole-cell patch clamp recordings were performed at room temperature using an EPC9 amplifier (HEKA Elektronik, Lambrecht/Pfalz, Germany). Transfected HEK293 cells were placed in a custom-made perfusion chamber that contained an extracellular solution ((in mM): 135 NaCl, 1.8 CaCl_2_, 1 MgCl_2_, 5.4 KCl, 10 glucose, and 10 HEPES, pH 7.35 adjusted with NaOH). The borosilicate glass patch pipettes were pulled on a DMZ universal puller (Zeitz Instruments, Martinsried, Germany), and filled with an intracellular solution ((in mM): 130 CsCl, 1 MgCl_2_, 5 EGTA, 15.62 CsOH and 10 HEPES, pH adjusted with HCl to 7.4). Serial resistance (Rs) was compensated for by 80%. Only cells that maintained a seal above 1 GΩ and had a maximum Rs of 10 MΩ were included. The macroscopic currents were sampled at 20 kHz and low pass filtered at 2.9 kHz. ClC-2 currents were activated by stepping the membrane potential to +20 to −140 in 20 mV decrements from a holding potential of 0 mV. Channels were activated for 5 s and subsequently stepped to +60 mV for 2 s to obtain a tail current. Channels were allowed to deactivate for 15 s between each sweep. Data were acquired in PatchMaster (v2x90 HEKA Elektronik). To construct the IV curve, the steady state current recorded at the end of the 5 s voltage step was plotted as a function of the clamp potential.

### *In vitro* transcription and *Xenopus laevis* oocyte injection

cRNA for *Xenopus laevis* oocyte injection was generated by *in vitro* transcription (T7 polymerase mMessage mMachine Kit, (Thermo Fisher Scientific, Ambion, Austin, TX, USA)) using linearized human wild-type or mutant ClC-2 cDNA. RNA quality and concentrations were assessed by gel electrophoresis and NanoDrop 2000 (Thermo Fisher Scientific, Ambion, Austin, TX, USA), respectively. *Xenopus laevis* oocytes were obtained from EcoCyte Bioscience (Castrop-Rauxel, Germany) and kept in ND96 medium (in mM: 4 KCl, 1.8 CaCl_2_, 1 MgCl_2_, 96 NaCl, HEPES, pH: 7.4) after arrival. We injected oocytes with 50 nl solution containing 2.5 ng ClC-2 WT, 2.5 ng ClC-2 MUT or 1.25 + 1.25 ClC-2 WT/ClC-2 MUT, respectively, using Nano-inject II (Drummond, Broomal, PA, USA). Oocytes were incubated for two days at 19 °C and then subjected to two-electrode voltage-clamp recordings.

### Two-electrode voltage-clamp

Two-electrode voltage-clamp (TEVC) recordings were performed using a Dagan CA-1B High-Performance Oocyte Clamp amplifier (Dagan, Minneapolis, MN, USA) in combination with an EPC-9 patch-clamp amplifier and PatchMaster software (Both HEKA Elektronik, Lambrecht/Pfalz, Germany). Oocytes were placed in a perfusion chamber under a continuous flow with ND96 solution at room temperature (21.5–23 °C). TEVC recording microelectrodes were pulled from borosilicate glass capillaries (Module Ohm, Herlev, Denmark) on a DMZ Universal Puller (Zeitz Instruments, Munich, Germany). The tip resistance of both microelectrodes was between 0.2–0.8 MΩ when filled with 3 M KCL. Currents through expressed channels were recorded in response to a step protocol (5 s depolarizations between 20 mV and −140 mV at 20 mV intervals, followed by a tail pulse to 60 mV. The results were confirmed in three different batches of oocytes with un-injected oocytes in parallel as a control.

Data were analyzed using Igor Pro (Wavemetrics, Lake Oswego, OR, USA) and GraphPad Prism 7 (GraphPad Software Inc., San Diego, CA, USA). I/V-curves were constructed as described for the patch clamp recordings. Plots of the voltage dependence of channel activation were generated by analyzing the peak tail current recorded at +60 mV. Peak tail currents were normalized to the maximum tail current recorded and plotted as a function of the preceding clamping potential. The mean normalized tail current was then fitted to a Boltzmann function:

$$\frac{I}{{I}_{max}}={I}_{min}+\frac{({I}_{max}-{I}_{min})}{1+{e}^{({V}_{0.5}-{V}_{m})/k}}$$

Where *I* is the current, *I*_*max*_ and *I*_*min*_ is the maximum and minimum tail current amplitude, *k* is the slope factor, *V*_*m*_ is the clamp potential, and *V*_*0*.*5*_ is the potential at 50% of the maximal tail current amplitude.

### Western blotting

Cell lysates were prepared from three independent transfections as previously described^44^. Protein concentrations were determined by Bradford protein assays according to the manufacturer’s instructions. Proteins were separated on 4–20% SDS-polyacrylamide gels by gel-electrophoresis, and transferred to an Immobilon-FL Polyvinylidene Difluoride (PVDF) membrane (Merck Millipore, Massachusetts, USA) by wet blotting techniques. Membranes were blocked in Odyssey® blocking buffer/Phosphate buffered saline 1:1 (LI-COR Biosciences, Nebraska, USA), and incubated with primary mouse anti-HA (1:1000) (Covance, New Jersey, USA) or rabbit anti-Lamin (1:1000) (Cell signaling, Massachusetts, USA) antibodies at 4 °C overnight. Membranes were incubated with IRDye ® 800CW donkey-anti-mouse IgG (1:10000) (LI-COR Biosciences) and IRDye ® 680CW donkey-anti-rabbit IgG (1:10,000) (LI-COR Biosciences). Fluorescently labeled bands were visualized by Odyssey CLx and quantified with Image Studio software (LI-COR Biosciences).

### Data analysis and statistics

Data analysis was performed in PatchMaster (v2x90 HEKA Elektronik), Igor pro version 4 and GraphPad Prism 8. Electrophysiological and qPCR data are presented as mean ± SEM. Statistical comparisons were evaluated by one-way ANOVA with Turkey’s multiple comparison correction for gene expression analysis and two-way ANOVA with a Sidak’s multiple comparison post test for I/V relationships. Effects on voltage dependence of activation was addressed using a Student t-test. Statistical significance of P-value: *(P < 0.05), **(P < 0.005), ***(P < 0.0005).

## Supplementary information

Supplementary Material.

## Data Availability

The datasets generated during and/or analyzed during the current study are available from the corresponding authors on reasonable request.
