# Supplementary material for: A Novel Loss-of-Function Variant in the Chloride Ion Channel Gene Clcn2 Associates with Atrial Fibrillation
Source: Sci Rep. 2020 Jan 29;10:1453. doi: 10.1038/s41598-020-58475-9 (PMC6989500; doi:10.1038/s41598-020-58475-9)
Supplement: Supplementary file 1 — Supplementary Material. [file 41598_2020_58475_MOESM1_ESM.pdf]

A NOVEL LOSS-OF-FUNCTION VARIANT IN THE CHLORIDE ION CHANNEL GENE *CLCN2* ASSOCIATES WITH ATRIAL FIBRILLATION

Thea Hyttel Hansen[1#], Yannan Yan[1], Gustav Ahlberg[1,2], Oliver Bundgaard Vad[1,2], Lena Refsgaard[2], Joana Larupa dos Santos[1], Nancy Mutsaers[1], Jesper Hastrup Svendsen[2,3], Morten Salling Olesen[1,2], Bo Hjorth Bentzen[1*], Nicole Schmitt[1*]

[1]Department of Biomedical Sciences, Faculty of Health and Medical Sciences, University of Copenhagen, Copenhagen, Denmark; [2]Laboratory for Molecular Cardiology, Department of Cardiology, The Heart Centre, Righospitalet, Copenhagen University Hospital, Denmark; [3]Department of Clinical Medicine, Faculty of Health and Medical Sciences, University of Copenhagen, Denmark

[#]Present address: ALK-Abelló A/S, 2970 Hørsholm, Denmark

[*]equally contributing last authors

Corresponding author: Nicole Schmitt, PhD

Dept. of Biomedical Sciences, Faculty of Health and Medical Sciences, University of Copenhagen, Mærsk Tower 07-9-36, Blegdamsvej 3B, DK-2200 Copenhagen N, Denmark; Phone: +45 3535327448; Fax: +45 35327555; E-mail: nschmitt@sund.ku.dk

CONTENTS

Supplementary Methods 3
  iPSC culture, differentiation, RNA extraction, cDNA synthesis 3
Supplementary Tables 5
  Supplementary Table S1. Clinical characteristics 5
  Supplementary Table S2. Sequencing coverage 6
  Supplementary Table S3. Rare or novel protein altering variants shared by affected family members 7
  Supplementary Table S4. Summary of number of annotations in each exon of family members 9
Supplementary Figures 10
  Supplementary Figure S1. Pathway analysis of human CLCN2 10
  Supplementary Figure S2. Pathway analysis of human CCDC15 11
  Supplementary Figure S3. Pathway analysis of human KCTD5 12

Supplementary Figure S4. Pathway analysis of human NEK1 13
Supplementary Figure S5. Pathway analysis of human PLCZ1 14
Supplementary Figure S6. Amino acid alignment of ClC-2 proteins 15
Supplementary Figure S7. Assessment of ClC-2 expression in induced pluripotent stem cell derived cardiomyocytes (iPSC-CM) 16
Supplementary Figure S8. Full-length western blot referring to Figure 5A 17
Supplementary References... 19

SUPPLEMENTARY METHODS

iPSC culture, differentiation, RNA extraction, cDNA synthesis

Induced pluripotent stem cells were cultured on matrigel® matrix (Corning, NY, USA) in TeSRTM-E8TM medium (StemCell Technologies, Vancouver, Canada) and split every 5-7 days by using standard stem cell culture procedures. Differentiated areas were removed and colonies were dissociated using gentle dissociation reagent (StemCell Technologies) and a cell scraper according to manufacturer's instructions. Two million cells were seeded per well in a 12-well plate coated with laminin-521 (Biolamina, Sundbyberg, Sweden). Differentiation was initiated four days after seeding by changing the media into RPMI 1640 supplemented with B27®-insulin (RPMI/B27) (both Life Technologies, Waltham, MA, USA). In the first 24 hours the media was supplemented with 12 µM of the GSK3 inhibitor CHIR99021 (Selleckchem, Munich, Germany) whereafter fresh RPMI /B27 was added to the cells (day one). On day three of differentiation half of the media was changed to fresh RPMI /B27 and 5 µM IWP2 (Tocris Bioscience, Bristol, UK), an inhibitor of Wnt processing and secretion, was added. Two days later the media was again changed and 1 µM retinoic acid was added to direct the cells towards an atrial-like phenotype[1]. After seven days and onwards the cells were maintained in media with insulin (RPMI 1640 supplemented with B27®+insulin (Life Technologies)). Within seven days beating areas in the well were evident. RNA was extracted from wells containing beating cells that were 32 or 203 days old counting from start of differentiation. RNA extraction was performed using the miRNeasy Mini Kit (Qiagen, Hilden, Germany) by following manufacturer's instructions. A QIAshredder (Qiagen) was used to homogenize the cells and a DNAse step was included (Qiagen). cDNA synthesis was performed using one µg RNA from 32 or 203 days old iPSC-CM. Reverse transcription was performed using the Precision nanoScriptTM 2 kit (Primer design, Chandler's Ford, UK) by following the manufacturer's instructions. A combination of oligo-dT and random nonamers were used as primers. For each RNA sample a reaction with no reverse transcriptase (-RT) was performed for later assessment of genomic DNA contamination.

For PCR, we used primers hCLC2-m.278-298 (forward: 5'-gaggaagctgctcggattcgc-3') and hCLC2-m.753-732 (reverse: 5'-caggtcagcccaatgaccttag-3'). As positive controls, we used cDNA from mammalian cells transfected with human ClC-2. Furthermore, we used primers sets for GAPDH (expected amplicon: 372 bp) and cardiac troponin T (cTnT, expected amplicon: 145 bp)

to assess cDNA quality of our iPCS-CM cDNA preparations. As negative controls, we used cDNA from ClC-1 transfected cells together with standard controls (no-template, no-enzyme).

SUPPLEMENTARY TABLES

Supplementary Table S1. Clinical characteristics

**Subject ID**	**Onset of symptoms (age)**	**Diagnosis of AF (age)**	**Type AF**	**Comorbidities (age of onset)**	**Echocardiogram**			
					**LAD**	**LVDd**	**LVEF (%)**	**Valvular abnormalities**
III-1	30	30	Paroxysmal	None	Normal	Normal	>55	Small, central mitral regurgitation
III-2	32	35	Persistent	Obesity Thyrotoxicosis (age 37)	Normal	Normal	>55	None
II-2	30	52	Paroxysmal*	Ischemic stroke (age 64), Hypertension (age 64), COPD (age 65)	Enlarged	Normal	>55	None

Abbreviations: AF, Atrial Fibrillation; COPD, Chronic obstructive pulmonary disease; LAD, Left atrial diameter; LVDd, Left ventricular diameter (diastolic); LVEF, Left ventricular ejection fraction. * = Patient developed permanent AF at age 66.

Supplementary Table S2. Sequencing coverage

Genotyping rate	Singeltons	TiTv	Mean	Median	bases above 10 reads (%)	bases above 20 reads (%)
0.988012	232	2.6051	90.06	76	97.8	93.9
0.987413	263	2.59392	101.08	85	98.1	95
0.988317	220	2.62816	93.34	78	98.2	94.7

TiTv, Transition to Transversion

Supplementary Table S3. Rare or novel protein altering variants shared by affected family members

**variantID**	**Gene**	**Effect**	**Impact**	**Transcript**	**AA change**	**CADD PHRED**	**D2K MAF**	**GERP score**
2:131098508_G/A	*CCDC115*	NS-C	Moderate	ENST00000437688	R130C	23.9	0.0002503	2.77
2:224831671_T/C	*MRPL44*	NS-C	Moderate	ENST00000258383	F307L	16.89	0	3.48
**3:184074821_TCACC/T**	***CLCN2***	**FS**	**High**	**ENST00000265593**	**QV347**	**34**	**0.0002503**	**0**
4:170477082_C/A	*NEK1*	SPD+I	High	ENST00000507142		32	0.0002503	5.66
4:185634100_G/A	*CENPU*	NS-C+ SP	Moderate	ENST00000281453	S229L	20.9	0	3.02
6:143858286_C/A	*PHACTR2*	NS-C	Moderate	ENST00000367584	S53R	23.1	0	2.32
10:105799226_G/A	*COL17A1*	NS-C	Moderate	ENST00000353479	P958L	23.4	0	3.71
12:18852779_C/G	*PLCZ1*	NS-C	Moderate	ENST00000266505	E375Q	26.5	0.0002503	5.88
13:39263071_C/A	*FREM2*	NS-C	Moderate	ENST00000280481	D530E	22.8	0	3.34
13:52952289_T/C	*THSD1*	NS-C	Moderate	ENST00000258613	R606G	17.5	0	-0.743
15:23686591_C/T	*GOLGA6L2*	NS-C	Moderate	ENST00000567107	R344Q	8.851	0	0
15:58957324_T/C	*ADAM10*	NS-C	Moderate	ENST00000260408	Q186R	22.9	0	5.6
16:2749840_T/C	*KCTD5*	NS-C	Moderate	ENST00000564195	V127A	25.7	0.0002503	5.2
16:30913430_C/T	*CTF1*	NS-C	Moderate	ENST00000279804	P59L	28.1	0	5.24
17:45925453_C/T	*SP6*	NS-C	Moderate	ENST00000342234	A115T	11.15	0	0.896

17:65942096_A/C	*BPTF*	NS-C	Moderate	ENST00000321892	Q2550H	12.01	0	-0.292
19:14080959_G/A	*RFX1*	NS-C	Moderate	ENST00000254325	T448M	26.2	0	5.41
19:47549436_C/T	*TMEM160*	NS-C	Moderate	ENST00000253047	G126R	15.53	0	3.32

AA, amino acid; CADD, Combined Annotation Dependent Depletion; D2K, 2000 Danish exomes; GERP, genomic evolutionary rate profiling; FS, frame shift; I, intron; MAF, minor allele frequency, NS-C, non_synonymous_coding; SP, splice_site_region; SPD, Splice_site_donor

Supplementary Table S4. Summary of number of annotations in each exon of family members

Sample ID	SNPs	Insertions	Deletions	Missense	Nonsense	Silent	frame shift
II-2	19.683	267	300	23.958	89	31.299	262
III-1	19.570	268	242	24.143	91	31.155	283
III-2	19.664	251	309	24.370	108	31.209	312

Note: Effect counts are on transcripts and therefore more than total of variants types (SNPs, Insertions, Deletions)

SUPPLEMENTARY FIGURES

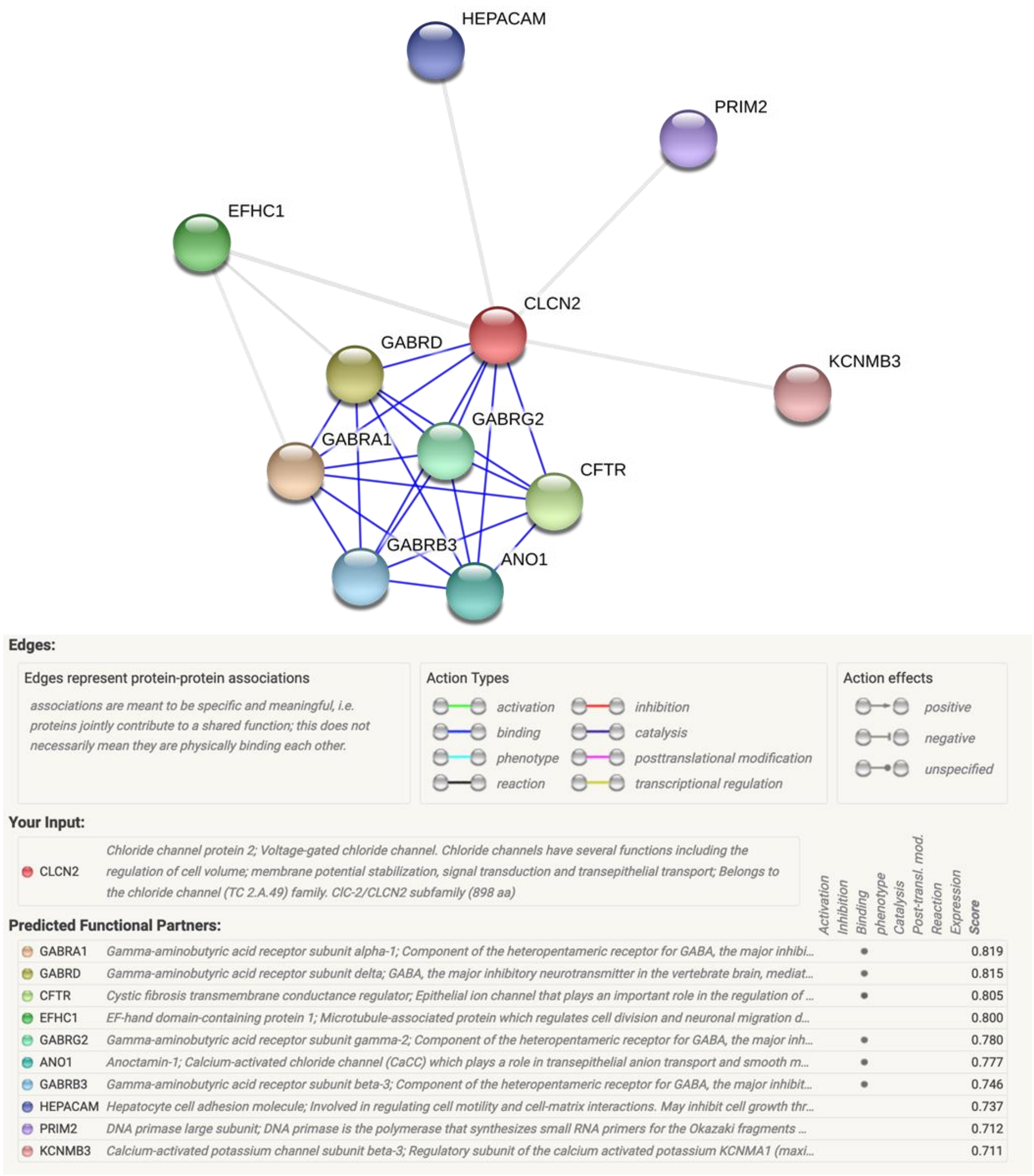

Supplementary Figure S1. Pathway analysis of human CLCN2. Pathway analysis of human ClC-2 was conducted using the free online tool STRING (https://string-db.org/)[2]. Protein interactions were filtered, and only protein interactions with medium confidence or more (confidence score > 0.400) were included.

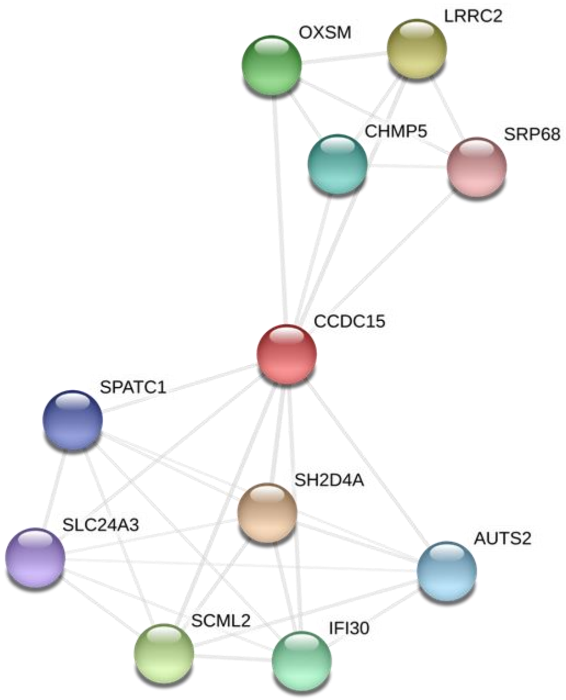

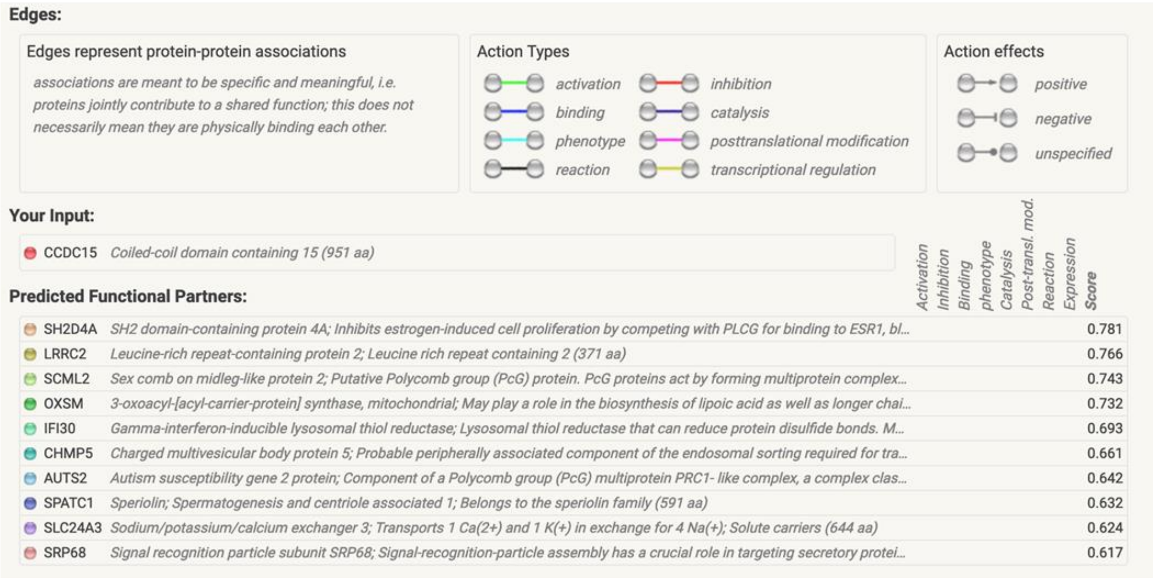

Edges:

Edges represent protein-protein associations

associations are meant to be specific and meaningful, i.e. proteins jointly contribute to a shared function; this does not necessarily mean they are physically binding each other.

Action Types

activation *inhibition*
binding *catalysis*
phenotype *posttranslational modification*
reaction *transcriptional regulation*

Action effects

positive
negative
unspecified

Your Input:

CCDC15 *Coiled-coil domain containing 15 (951 aa)*

Predicted Functional Partners:

		Activation	Inhibition	Binding	phenotype	Catalysis	Post-transl. mod.	Reaction	Expression	Score
SH2D4A	*SH2 domain-containing protein 4A; Inhibits estrogen-induced cell proliferation by competing with PLCG for binding to ESR1, bl...*									0.781
LRRC2	*Leucine-rich repeat-containing protein 2; Leucine rich repeat containing 2 (371 aa)*									0.766
SCML2	*Sex comb on midleg-like protein 2; Putative Polycomb group (PcG) protein. PcG proteins act by forming multiprotein complex...*									0.743
OXSM	*3-oxoacyl-[acyl-carrier-protein] synthase, mitochondrial; May play a role in the biosynthesis of lipoic acid as well as longer chai...*									0.732
IFI30	*Gamma-interferon-inducible lysosomal thiol reductase; Lysosomal thiol reductase that can reduce protein disulfide bonds. M...*									0.693
CHMP5	*Charged multivesicular body protein 5; Probable peripherally associated component of the endosomal sorting required for tra...*									0.661
AUTS2	*Autism susceptibility gene 2 protein; Component of a Polycomb group (PcG) multiprotein PRC1- like complex, a complex clas...*									0.642
SPATC1	*Speriolin; Spermatogenesis and centriole associated 1; Belongs to the speriolin family (591 aa)*									0.632
SLC24A3	*Sodium/potassium/calcium exchanger 3; Transports 1 Ca(2+) and 1 K(+) in exchange for 4 Na(+); Solute carriers (644 aa)*									0.624
SRP68	*Signal recognition particle subunit SRP68; Signal-recognition-particle assembly has a crucial role in targeting secretory protei...*									0.617

Supplementary Figure S2. Pathway analysis of human CCDC15. Pathway analysis was performed as described above (Supplementary Figure S1).

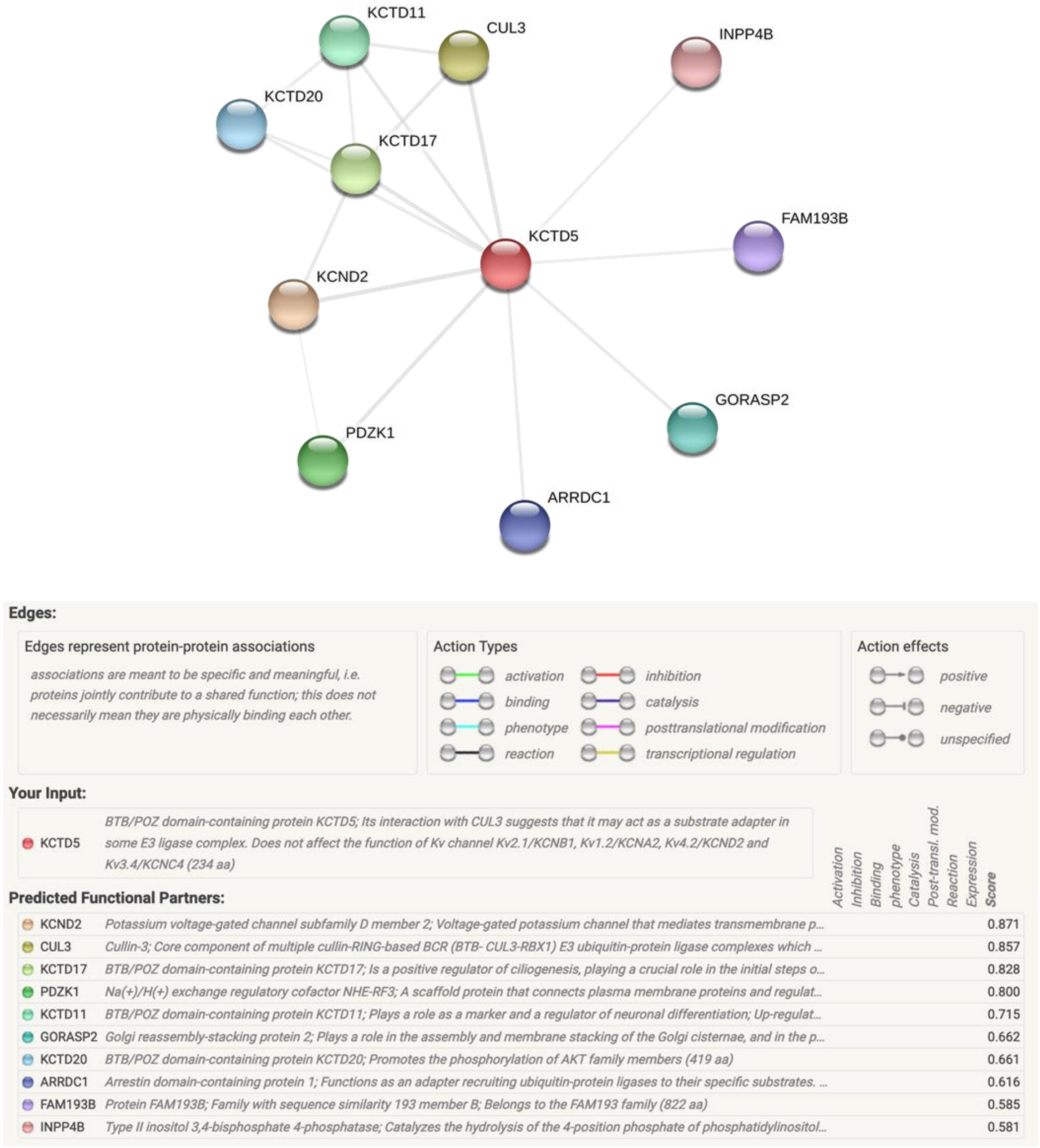

Supplementary Figure S3. Pathway analysis of human KCTD5. Pathway analysis was performed as described above (Supplementary Figure S1).

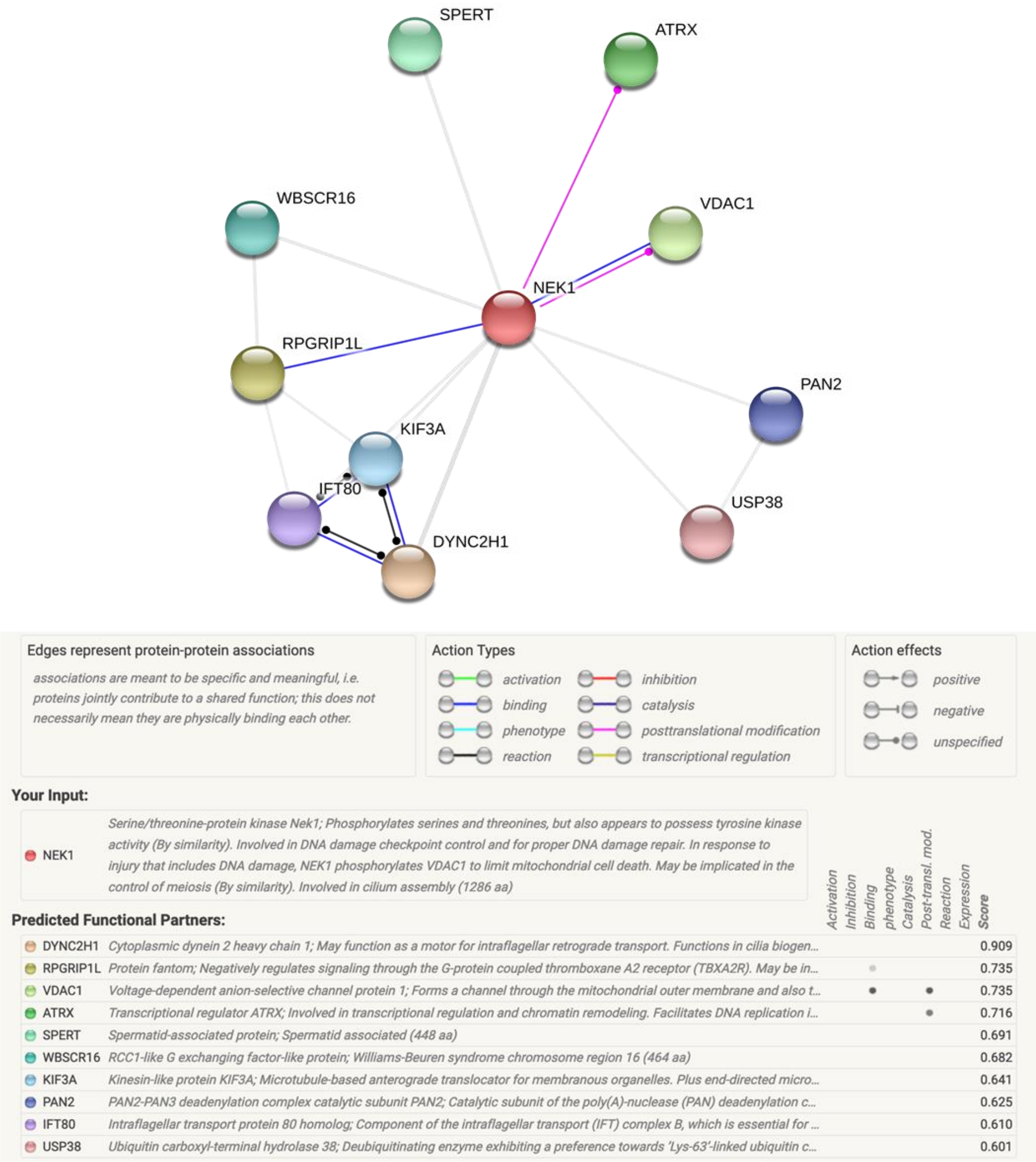

Supplementary Figure S4. Pathway analysis of human NEK1. Pathway analysis was performed as described above (Supplementary Figure S1).

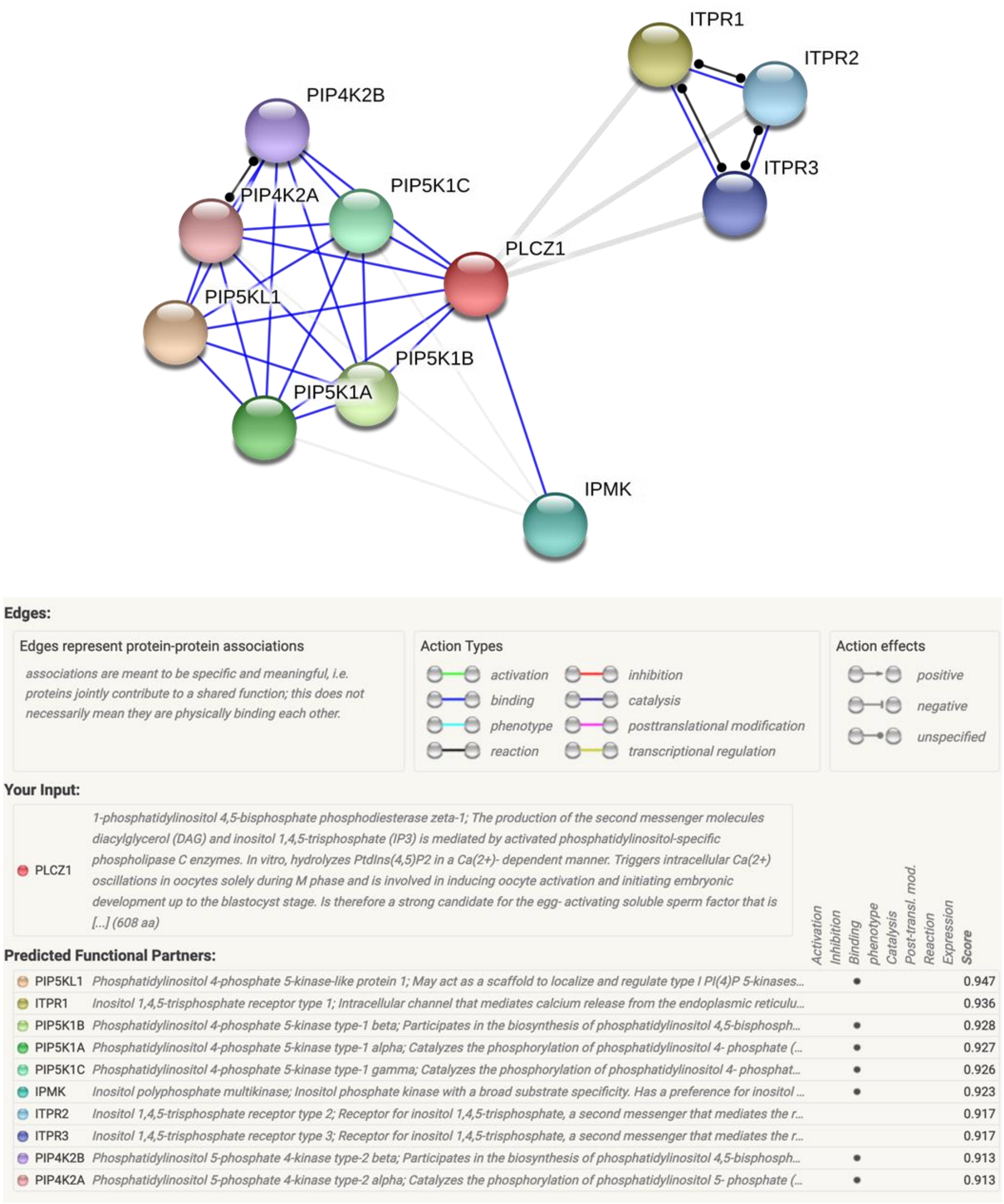

		Activation	Inhibition	Binding	phenotype	Catalysis	Post-transl. mod.	Reaction	Expression	Score
PIP5KL1	Phosphatidylinositol 4-phosphate 5-kinase-like protein 1; May act as a scaffold to localize and regulate type I PI(4)P 5-kinases...			•						0.947
ITPR1	Inositol 1,4,5-trisphosphate receptor type 1; Intracellular channel that mediates calcium release from the endoplasmic reticulu...									0.936
PIP5K1B	Phosphatidylinositol 4-phosphate 5-kinase type-1 beta; Participates in the biosynthesis of phosphatidylinositol 4,5-bisphosph...			•						0.928
PIP5K1A	Phosphatidylinositol 4-phosphate 5-kinase type-1 alpha; Catalyzes the phosphorylation of phosphatidylinositol 4- phosphate (...			•						0.927
PIP5K1C	Phosphatidylinositol 4-phosphate 5-kinase type-1 gamma; Catalyzes the phosphorylation of phosphatidylinositol 4- phosphat...			•						0.926
IPMK	Inositol polyphosphate multikinase; Inositol phosphate kinase with a broad substrate specificity. Has a preference for inositol ...			•						0.923
ITPR2	Inositol 1,4,5-trisphosphate receptor type 2; Receptor for inositol 1,4,5-trisphosphate, a second messenger that mediates the r...									0.917
ITPR3	Inositol 1,4,5-trisphosphate receptor type 3; Receptor for inositol 1,4,5-trisphosphate, a second messenger that mediates the r...									0.917
PIP4K2B	Phosphatidylinositol 5-phosphate 4-kinase type-2 beta; Participates in the biosynthesis of phosphatidylinositol 4,5-bisphosph...			•						0.913
PIP4K2A	Phosphatidylinositol 5-phosphate 4-kinase type-2 alpha; Catalyzes the phosphorylation of phosphatidylinositol 5- phosphate (...			•						0.913

Supplementary Figure S5. Pathway analysis of human PLCZ1. Pathway analysis was performed as described above (Supplementary Figure S1).

```
human   288 IFRVLAVWNRDEETITALFKTRFRLDFPFDLQELPAFAVIGIASGFGGALFVYLNRKIVQVMRKQKTINRFLMRKRLLFP  367
MUT     288 IFRVLAVWNRDEETITALFKTRFRLDFPFDLQELPAFAVIGIASGFGGALFVYLNRKIVQCGSRKPSIASS.
monkey  288 IFRVLAVWNRDEETITALFKTRFRLDFPFDLQELPAFAVIGIASGFGGALFVYLNRKIVQVMRKQKTINRFLMRKRLLFP  367
pig     312 IFRVLAVWNRDEETITALFKTRFRLDFPFDLQELPAFAVIGIASGFGGALFVYLNRKIVQVMRKQKTINRFLMKKRLLFP  391
mouse   303 IFRVLAVWNRDEETITALFKTRFRLDFPFDLQELPAFAVIGIASGFGGALFVYLNRKIVQVMRKQKTINRFLMRKRLLFP  382
                                              J                             K

human   368 ALVTLLISTLTFPPGFGQFMAGQLSQKETLVTLFDNRTWVRQGLVEELEPPSTSQAWNPPRANVFLTLVIFILMKFWMSA  447
monkey  368 ALVTLLISTLTFPPGFGQFMAGQLSQKETLVTLFDNRTWVRQGLVEELEPPSTSQAWNPPRANVFLTLVIFILMKFWMSA  447
pig     392 ALVTLLISTLTFPPGFGQFMAGQLSQKETLVTLFDNRTWVRQGLMEEVEPPGTSQAWSPPRANVFLTLVIFILMKFWMSA  471
mouse   383 ALVTLLISTLTFPPGFGQFMAGQLSQKETLVTLFDNRTWVRQGLVEDLELPSTSQAWSPPRANVFLTLVIFILMKFWMSA  462
                                       L                             M

human   448 LATTIPVPCGAFMPVFVIGAAFGRLVGESMAAWFPDGIHTDSSTYRIVPGGYAVVGAAALAGAVTHTVSTAVIVFELTGQ  527
monkey  448 LATTIPVPCGAFMPVFVIGAAFGRLVGESMAAWFPDGIHTDSSTYRIVPGGYAVVGAAALAGAVTHTVSTAVIVFELTGQ  527
pig     472 LATTIPVPCGAFMPVFVIGAAFGRLVGESMAAWFPDGIHTDSSTYRIVPGGYAVVGAAALAGAVTHTVSTAVIVFELTGQ  551
mouse   463 LATTIPVPCGAFMPVFVIGAAFGRLVGESMAAWFPDGIHTDSSTYRIVPGGYAVVGAAALAGAVTHTVSTAVIVFELTGQ  542
                      N                              O
```

Supplementary Figure S6. Amino acid alignment of ClC-2 proteins. Alignment of orthologous proteins from human (*Homo sapien*s, GenBank Acc. No. NP_004357.3), monkey (*Pan troglodytes*, XP_016797900.1), pig (*Sus scrofa*, XP_013837709.1), and mouse (*Mus musculus*, XP_017172351.1) in the regions surrounding the predicted transmembrane domains (boxed in grey, segments J to O, see Figure 1) affected by the frame shift mutation. The position of the deletion del1041-1044 in the corresponding nucleotide sequence is indicated by an arrow. The putative sequence of the mutant protein (MUT) is given below the wild-type sequence; the resulting putative protein will be truncated in segment J.

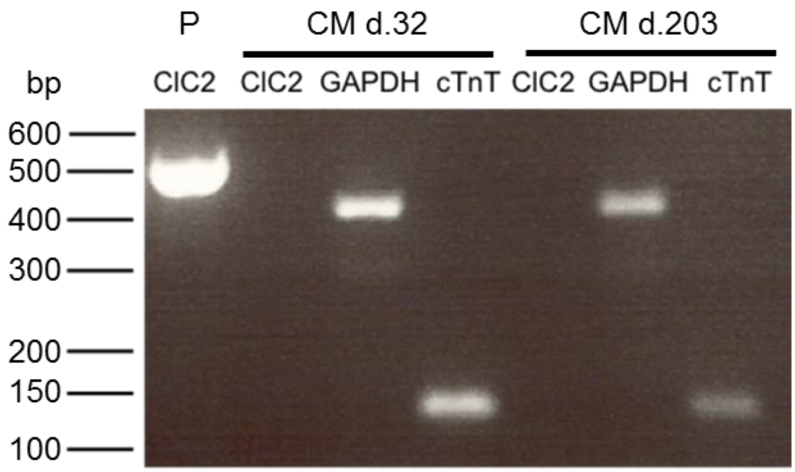

Supplementary Figure S7. Assessment of ClC-2 expression in induced pluripotent stem cell derived cardiomyocytes (iPSC-CM). End-point reverse transcription PCR employing 32 (CM d.32) or 203 (CM d.203) days old iPSC-CM, respectively, did not reveal expression of the channel. As possible control, we used cDNA produced from hClC-2 expressing HEK293 cells (P). Cardiac troponin (cTNT, expected amplicon: 145 bp) and housekeeping gene GAPDH (expected amplicon: 372 bp) served as controls for the integrity of cDNA produced from iPSC-CM. Separation of PCR fragments on 2% (w/V) agarose gels containing 0,5 µg/mL ethidiumbromide, visualized with UV light. Size marker is shown to the left. The application of ClC-2 specific primers resulted in a product at the expected length of 475 bp in the positive control, but did not result in any signals in either of the iPSC-CM samples. Negative controls did not show any contaminations (data not shown).

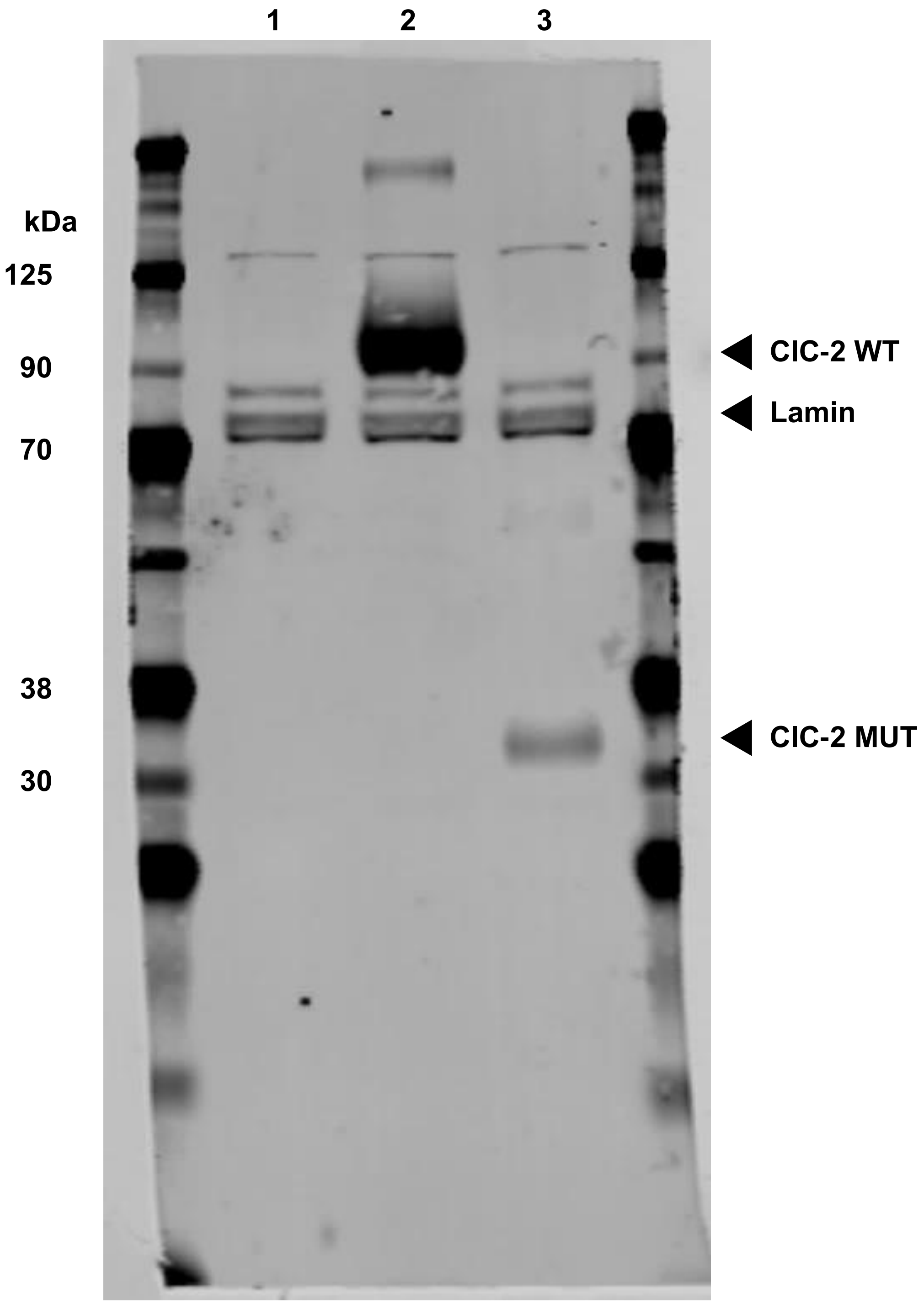

Supplementary Figure S8. Full-length western blot referring to Figure 5A. Representative western blot of protein extracts from HEK293 cells transfected with empty plasmid (lane 1), ClC-2 WT (lane 2) or ClC-2 MUT (lane 3). Lamin was used as a loading control. Bands representing ClC-2 or Lamin proteins are indicated by arrows.

SUPPLEMENTARY REFERENCES

1. Devalla, H. D. *et al.* Atrial-like cardiomyocytes from human pluripotent stem cells are a robust preclinical model for assessing atrial-selective pharmacology. *EMBO Mol. Med.* (2015) doi:10.15252/emmm.201404757.
2. Szklarczyk, D. *et al.* STRING v11: protein-protein association networks with increased coverage, supporting functional discovery in genome-wide experimental datasets. *Nucleic Acids Res.* **47**, D607–D613 (2019).
